# Supplementary material for: Phylogenetic and taxonomic revisions of Jurassic sea stars support a delayed evolutionary origin of the Asteriidae
Source: PeerJ. 2024 Oct 31;12:e18169. doi: 10.7717/peerj.18169 (PMC11531740; doi:10.7717/peerj.18169)
Supplement: Supplemental Information 2 [file peerj-12-18169-s002.docx]

**Supplementary materials S1:** Character list

Characters 30, 31, have been modified from the original character matrix, characters 117 and 118 have been added from the original character matrix by Fau & Villier (2020).

1. Number of arms: (0) 5 arms; (1) 6 to 16 arms; (2) 17 to 50 arms.
2. Position of the peristome: (0) actinostome; (1) deeply sunken actinostome.
3. Partial fusion of the oral frame ossicles (orals, first ambulacrals and odontophores) forming a rigid ring: (0) absent; (1) present.
4. Odontophore, *crater*: (0) absent; (1) present.
5. Odontophore, butterfly shape: (0) absent; (1) present.
6. Odontophore: (0) longer than wide; (1) wider than long.
7. Odontophore: (0) distal part approximately the same size or wider than the proximal part; (1) proximal part wider than the distal part.
8. Odontophore, articulation areas *poda* and *doda*: (0) fused; (1) separated, but on the same surface; (2) physically separated by a lateral notch.
9. Articulation area *doda* of the odontophore linked to: (0) orals only; (1) both orals and first ambulacrals; (2) first ambulacrals only.
10. First ambulacral, *furrow*: (0) absent; (1) present.
11. First ambulacral, ratio between the length and the height of the distal process: (0) < 0.9; (1) between 0.9 and 1.7; (2) > 1.7.
12. First ambulacral, ratio between the length and the height of the proximal process: (0) < 2; (1) between 2 and 4; (2) between 4 and 6; (3) > 6.
13. First ambulacral, ratio between the height of the proximal and distal process: (0) < 2; (1) >2.
14. First ambulacral, position of the articulation with the odontophore *doda*: (0) on the base of *distal process*; (1) on the *shaft*/*head* of the distal process.
15. First ambulacral, *distal process*: (0) distal process projecting distally and forming an angle with the head; (1) distal process and distal side of the head on the same line.
16. First ambulacral, orientation of the *procoa* and *dicoa*: (0) nearly parallel; (1) not parallel.
17. First ambulacral, angle between the *proximal process* and the proximal edge of the *head*: (0) acute angle; (1) right or obtuse angle.
18. Orals, surface of the articulation *iioa*: (0) made of smooth stereom; (1) surface not smooth, sometimes made of imperforate stereom.
19. Orals, teeth on the interoral articulation *iioa*: (0) absent; (1) present.
20. Orals, position of the articulation *iioa* compare to the muscle insertion *aciim*: (0) *iioa* in actinal position; (1) *iioa* in abactinal position; (2) *aciim* divided in two around the *iioa*.
21. Orals, proximal angle of the *body*: (0) smooth angle, more or less 90°; (1) sharp angle, less than 70°.
22. Orals, position of the *rvg*: (0) in proximal position compare to the *abiim*; (1) in abactinal position compare to the *abiim*.
23. Orals, *rvg*: (0) very shallow, almost invisible; (1) well defined.
24. Orals, contact with the second ambulacral along the distal edge: (0) absent; (1) present.
25. Orals, number of enlarged spine bases (*round* *pustules* or *keyhole pustules*): (0) 1 to 3; (1) 4 and more.
26. Ambulacral, ratio between the *length* and the height of the ossicle: (0) low (< 4.5); (1) average (4.5 < X < 9); (2) high (> 9).
27. Ambulacral, ratio between the *teeth length* and the *minimal body length*: (0) < 1; (1) 1 =< X < 2, rod shape; (2) 1 =< X < 2, hourglass shape.
28. Ambulacral, abactinal bending of the ambulacral ossicles: (0) absent, the abactinal edge is rather straight; (1) abactinal edge slightly depressed in its mid part; (2) strongly arched.
29. Ambulacral, extension on the *head* of the *lim* muscle insertion: (0) *lim* short (less than 40% of the ossicle height); (1) *lim* long, more than 40% of the ossicle height and finishing under the *actam*.
30. Ambulacral, proximal tilting of the ambulacral crest: (0) absent; (1) present.
31. Ambulacral, proximal tilting of the ambulacral crest: (0) angle smaller than 45°; (1) angle greater than 45°.
32. Ambulacral, ambulacral’s *head*: (0) symmetrical; (1) asymmetrical, longer on the proximal side.
33. Ambulacral, furrow: (0) absent; (1) present, but irregular; (2) present, well-marked.
34. Ambulacral, abactinal surface of the shaft: (0) stereom undifferentiated; (1) glassy trabeculae present.
35. Ambulacral, proximal and distal extension of the ambulacral base (wings) for the muscles dadam and padam: (0) absent; (1) present.
36. Ambulacral, articulation area with the superambulacrals (sa): (0) absent; (1) bump present, but undifferentiated stereom; (2) differentiated.
37. Superambulacrals: (0) absent; (1) present, but reduced; (2) present.
38. Adoral carina: (0) absent; (1) present.
39. Adoral carina: (0) short, one to three adjoining adambulacrals present; (1) long, more than three adambulacrals involved.
40. Adoral carina, teeth on the interradial surface of the adambulacrals of the adoral carina: (0) absent; (1) present.
41. Adoral carina, number of spines on the adambularals of the adoral carina: (0) same number of spines as the other adambulacrals; (1) fewer spines than on other adambulacrals.
42. Adambulacrals, proximo-distal compression of the adambulacral ossicles: (0) not compressed (width = > length); (1) strongly compressed (width < length).
43. Adambulacrals, height: (0) ossicles higher than width; (1) ossicles wider than high.
44. Adambulacrals, relative size of the muscle insertion *dadam* and *padam*: (0) smaller than the muscle insertion *padam*; (1) approximately the same size as the muscle insertion *padam*.
45. Adambulacrals, articulation *dada*: (0) with two distinct surfaces; (1) with confluent surfaces.
46. Adambulacrals, ledge formed by the articulation *dada*: (0) rather concave; (1) rather flat or convex.
47. Adambulacrals, crest between the muscle insertion *padam* and *dadam*: (0) absent; (1) present.
48. Adambulacrals, adambulacrals with adaradial extension (carinate adambulacrals): (0) absent; (1) alternatively carinate and non-carinate adambulacrals.
49. Adambulacrals, number of adambulacral spines: (0) constant; (1) variable on adambulacrals of similar size.
50. Adambulacrals, number of primary spines: (0) 1 to 4; (1) 4 and more.
51. Adambulacrals, number of secondary spines: (0) absent; (1) present.
52. Adambulacrals, rows of spines oriented lengthwise (furrow spines): (0) absent; (1) present.
53. Adambulacrals, shape of the spines: (0) conical or cylindrical; (1) with a flattened extremity.
54. Adambulacrals, attachment of the straight pedicellariae: (0) on the adambulacrals; (1) on the spines.
55. Actinals: (0) absent or reduced, loose in dermal tissues; (1) present.
56. Actinals, number of rows: (0) only a few plates, two or three plates per arm; (1) 1 to 3 rows; (2) 4 and more.
57. Actinals: (0) non-overlapping one to each other; (1) overlapping like roof tiles.
58. Actinals, primary spines: (0) absent; (1) present.
59. Actinals, number of primary spines: (0) only one spine; (1) 2 or 3 spines; (2) 4 spines and more.
60. Actinals spines: (0) long and slender; (1) short and stout.
61. Actinals: wreath organs: (0) absent; (1) present.
62. Actinals, secondary spines: (0) absent; (1) present.
63. Marginals, number of rows: (0) only one row; (1) two rows.
64. Marginals, intermarginals: (0) absent; (1) present.
65. Inferomarginals, pitting of the internal face: (0) absent; (1) one pit; (2) two pits.
66. Inferomarginals, interbrachial wall composed of actinals and inferomarginals: (0) absent; (1) present.
67. Inferomarginals, number of primary spines: (0) one spine; (1) 2 or 3 spines; (2) 4 spines and more.
68. Inferomarginals, round pustule: (0) absent; (1) present.
69. Inferomarginals, keyhole pustule: (0) absent; (1) present.
70. Inferomarginals, secondary spines attached on bump: (0) absent; (1) present.
71. Inferomarginals, shape: (0) cruciform shape; (1) inverse T shape; (2) block-like.
72. Inferomarginals, spine tip: (0) conical or circular; (1) flattened.
73. Inferomarginals, spines: (0) similar to the superomarginal spines; (1) thicker and/or longer than the superomarginal spines.
74. Superomarginals, glassy tubercules or microlens arrays: (0) absent; (1) present.
75. Superomarginals, shape: (0) cruciform; (1) falciform; (2) block-like.
76. Superomarginals, intercalary ossicles: (0) absent; (1) present.
77. Superomarginals, shape, size of the abactinal and actinal lobes: (0) abactinal and actinal lobes approximately the same length; (1) actinal lobe longer (abactinal lobe reduced).
78. Superomarginals, number of primary spines: (0) only one spine; (1) 2 or 3 spines; (2) 4 spines and more.
79. Abactinals, number of abactinals separating the carinal from the marginal plate rows: (0) a single series; (1) two series or more.
80. Abactinals, degree of plate differentiation: (0) plates undifferentiated; (1) at least two level of plates.
81. Abactinals, structure of the wall skeleton: (0) compact, with adjoining or overlapping plates; (1) reticulate mesh; (2) reduced.
82. Abactinals, intercalary inter-arc ossicles: (0) absent; (1) present.
83. Abactinals, primary spine attached on pustules: (0) absent; (1) present.
84. Abactinal, spines: wreath organs: (0) absent; (1) present.
85. Abactinals: (0) flat or slightly arched; (1) strongly arched.
86. Carinals: (0) absent; (1) present.
87. Carinals, continuity of the ossicle row: (0) continuous; (1) non-continuous.
88. Carinals, row axis: (0) straight; (1) zigzag.
89. Carinals, articular lobes: (0) well-formed; (1) reduced.
90. Carinals, number of articular facets: (0) up to 4 articular facets; (1) more than 4 articular facets.
91. Carinals, number of primary spines: (1) 2 or 3 spines; (2) 4 spines and more.
92. Carinals, wreath organ on primary spines: (0) absent; (1) present.
93. Carinals, secondary spines: (0) absent; (1) present.
94. Carinals, glassy tubercles: (0) absent; (1) present.
95. Wall skeleton, primary spines (spines on actinals, marginals, abactinals and carinals): (0) long; (1) short.
96. Wall skeleton, primary spines (spines on actinals, marginals, abactinals and carinals): (0) slender; (1) stout.
97. Wall skeleton spines, ornamentation: (0) absent; (1) present.
98. Wall skeleton spines, bifid: (0) absent; (1) present.
99. Wall skeleton spines, stereom: (0) undifferentiated stereom; (1) differentiated stereom with glassy trabeculae.
100. Forcipulate pedicellariae: (0) absent; (1) present.
101. Straight pedicellariae: (0) absent; (1) present.
102. Straight pedicellariae, felipedal (Clark and Downey 1992): (0) absent; (1) present.
103. Straight pedicellariae, valve length relative to the width of the basal piece: (0) short or equal size; (1) 1.5 to 2 times longer.
104. Straight pedicellariae, location: (0) present only on the actinal side; (1) present on the whole body.
105. Crossed pedicellariae: (0) absent; (1) present.
106. Crossed pedicellariae, rows of distal teeth: (0) up to 3; (1) 3 or more.
107. Crossed pedicellariae, differentiated teeth (canines): (0) absent; (1) present.
108. Crossed pedicellariae, shape: (0) common shape; (1) long and slender.
109. Crossed pedicellariae, diastema between the distal teeth and the median teeth: (0) absent; (1) present, curved; (2) present, straight.
110. Crossed pedicellariae, medial projection: (0) absent; (1) present.
111. Crossed pedicellariae, wreath organ: (0) absent, random distribution of the crossed pedicellariae; (1) present.
112. Disc, abactinals (all plate except madreporite, radials and interradials): (0) thick, opaque; (1) thin, translucent.
113. Disc, glassy tubercles on radials and interradials: (0) absent; (1) present.
114. Madreporite: (0) isolated; (1) embedded in a special cavity of the adjoining interradial; (2) fused with an interradial.
115. Terminal, proximal notch: (0) absent; (1) present.
116. Terminal, glassy tubercles: (0) absent; (1) present.
117. Actinals swollen primary spine base: (0) absent; (1) present.
118. Marginals swollen primary spine base : (0) absent; (1) present.
119. Disc, radials overlapping the interradials: (0) absent; (1) present.
120. Actinals: keyhole spine attachment: (0) absent; (1) present.
